# Effect of blood donor characteristics on transfusion outcomes: a protocol for systematic review and meta-analysis

**DOI:** 10.1186/2046-4053-3-28

**Published:** 2014-03-20

**Authors:** Michaël Chassé, Shane W English, Lauralyn McIntyre, Greg Knoll, Nadine Shehata, Alan Forster, Kumanan Wilson, Carl van Walraven, Alan Tinmouth, Dean A Fergusson

**Affiliations:** 1 Clinical Epidemiology program, Ottawa Hospital Research Institute, 501 Smyth Road, Box 201B, Ottawa, ON K1H 8L6, Canada; 2 Mount Sinai Hospital, 600 University Avenue, Toronto, ON M5G 1X5, Canada; 3 Ottawa Hospital, Civic Campus, 1053 Carling Avenue, Box 684, Ottawa, ON K1Y 4E9, Canada; 4 Department of Medicine, University of Ottawa and Ottawa Hospital Research Institute, 501 Smyth Road, Box 201B, Ottawa, ON K1H 8L6, Canada; 5 Clinical Epidemiology Program, Box 201B, Ottawa Hospital Research Institute, 1053 Carling Avenue, Ottawa, ON K1Y 4E9, Canada

## Abstract

**Background:**

Optimal selection of blood donors is of paramount importance in ensuring the safety of blood products. The current selection process is concerned principally with the safety of the blood donor and the safety of the patient that receives the blood. Recent evidence suggests that the characteristics of the donor may affect transfusion outcomes for the recipient.

**Methods:**

We will conduct a systematic review of the association between major blood donor characteristics and red blood cell (RBC) transfusion outcomes. The primary objective is to assess the association of blood donor characteristics and the risk of adverse short-term and long-term clinical outcomes after RBC transfusion. We will search MEDLINE, EMBASE, Cochrane Central databases, as well as perform manual searches of top transfusion medical journals for prospective and retrospective studies. Study characteristics will be reported and the methodological quality of studies will be assessed. When appropriate, we will provide pooled odds ratio with 95% confidence intervals of the effect estimates, study clinical heterogeneity using pre-defined sensitivity and subgroup analyses, and study statistical heterogeneity using the I^2^ test.

**Discussion:**

The results of this systematic review will provide an evidence base regarding the potential clinical effects of donor characteristics on transfusion recipients to better guide policy and clinical practice. The evidence gathered from this review will also identify strengths and weaknesses of published studies regarding donor characteristics and transfusion outcomes and will identify knowledge gaps to inform future research in this field of transfusion medicine.

**Trial registration:**

PROSPERO Registration Number: CRD42013006726

## Background

Transfusion of blood products is a necessary, lifesaving intervention. More than 85 million units of red blood cells (RBCs) are collected and transfused every year worldwide [1]. RBCs are given to increase oxygen delivery to tissues and used across a variety of medical and surgical situations. For instance, approximately 30% of critical care patients, and more than 50% of cardiac surgery patients, will receive a transfusion during their hospital stay [2,3].

Importantly, the use of RBC transfusion is not without risks, including infectious and immunologic. These risks can be reduced by a variety of measures, including donor questionnaires that assess risk, infectious disease blood screening, and other quality measures already implemented by blood agencies [4,5]. An important aim of current measures in reducing risks for patients is to better select blood donors. These measures have been shown to be efficient in improving transfusion outcome. For example, better screening of donors and microbiological testing of the blood products have led to the reduction of transfusion-related infections as low as 1 in 8 million for HIV, 1 in 6.7 million for hepatitis C virus and 1 in 1.7 million for hepatitis B virus [6]. More recently, female sex, a history of pregnancy and the presence of anti-leukocytes antibodies in blood products have been associated with the risk of transfusion-related acute lung injury, which is the current leading cause of mortality after transfusion. These findings have led directly to successful interventions (collection of male-only plasma for example), with a subsequent decrease in the occurrence of transfusion-related acute lung injury in Canada [7,8]. Finally, red blood cell units have important biologic variability due to the fact that each unit is collected from an individual volunteer donor. This variability and the variability of interactions between donor and recipient may affect the clinical efficacy and safety of a blood transfusion. Although many measures are undertaken to select blood donors based on their clinical characteristics, there are no systematic reviews evaluating the impact of donor characteristics on transfusion recipient morbidity and mortality.

### Limitations of evidence

Notably, the medical literature is extensive regarding the impact of donor characteristics on outcome of solid organ and bone marrow transplanted patients. These characteristics are used routinely to determine if a specific organ may be suitable for transplantation. Patient characteristics are also used to select the best recipient for a particular organ and to optimize follow-up of transplanted patients when some characteristics from the donor are not optimal. In contrast, when it comes to blood transfusion (which is similar to the transplantation of a different organ) there is a paucity of data regarding the impact of donor characteristics on outcome. We suggest the following evidence to support our rationale to examine the effect of donor characteristics on transfusion outcomes: 1) literature for solid organ transplantation demonstrates the effect of age and gender on recipient outcomes [9-13]; 2) there are immunological modifications that are associated with age such as the increase in memory T cells from naïve T cells [13]; 3) decreased tolerance of the immune system with age[13]; 4) in platelet transfusion, allergic transfusion reactions seem to be associated more with recipient and donor factors than with product attributes [14]; and 5) immunological phenomena in the donor such as the anti-leukocyte antibodies (anti-human-leukocyte antibodies or anti-neutrophil antibodies) that occur after pregnancies (for example gender effect on transfusion-related acute lung injury) and the successful interventions (collection of male-only plasma) to reduce the occurrence of adverse events and improve the safety of RBC transfusion [7,8]. The biological plausibility, clinical evidence in solid organ transplant, and current policies that select donors based on their characteristics mandates a systematic review to provide a rigorous evidence base for the potential effects of donor characteristics and their impact on outcome.

### Research objectives

The primary objective of this systematic review is to evaluate the association of blood donor characteristics (for example age, gender, ABO blood type) and the risk of adverse short-term and long-term clinical outcomes after RBC transfusion in recipients. Our secondary objectives are: 1) to assess the risk of bias of studies informing current policies for selection of blood donors on their characteristics; and 2) to identify knowledge gaps and potential future research directions in the selection of blood donors based on their characteristics.

## Methods/Design

The methods to be used for this systematic review will follow strict methodological standards based on the Cochrane Collaboration recommendations [15]. Our approach will include, in addition to our research goals and objectives, a thorough process for study identification and selection, a descriptive quality assessment of included studies, and a data analysis, and interpretation.

### Search strategy

We will develop a comprehensive and systematic search strategy with an information specialist trained in the conduct of systematic reviews. We will use MeSH (or EMTREE equivalent) terms in addition with free text terms representing the included population, exposure to RBC and donor characteristics and outcomes, to be sensitive and inclusive (Additional file 1). We will search MEDLINE database, EMBASE database and Cochrane Central databases (that include the Cochrane Database of Systematic Reviews, the Cochrane Central Register of Controlled Trials, the Cochrane Methodology Register, the Database of Abstracts of Reviews of Effects, the Health Technology Assessment Database and the NHS Economic Evaluation Database) from inception and updated until authors are ready for final abstraction of selected references. Our search strategy will have no language restrictions. Reference lists of published narrative reviews, systematic reviews, and eligible studies will be searched for additional references. We will finally perform manual screening of published articles since the year 2000 of five journals in the field of transfusion medicine, according to the 2012 Thomson Reuters’ impact factor and from expert opinion of the most clinically relevant journals in the field (*Blood*, *Transfusion Medicine Review*, *British Journal of Hematology*, *Vox Sanguinis* and *Transfusion*).

### Study screening and inclusion

Using the results of our comprehensive search strategy, we will obtain title and abstract from all references. If an abstract is not available, full text will be obtained unless the title is clearly irrelevant. From abstracts and titles, a screening process will be performed by two independent reviewers for each reference using the inclusion and exclusion criteria below. Full text copies of relevant reports will then be obtained for independent analysis by two reviewers for final inclusion decision. Two independent reviewer will then collect the required data from selected studies using a piloted electronic form. Disagreements will be resolved by consensus and by consultation with a third independent reviewer when needed.

### Inclusion criteria for review

#### Study type

We will include both observational studies (including cross-sectional and cohort studies) and interventional studies. Our aim in synthesizing all available evidence is to provide not only estimates of association but also the clinical and methodological quality of the evidence and identify important gaps in the evidence.

#### Population

The aim of RBC transfusions is to improve oxygen delivery to tissues. Such is the case for patients with active bleeding of any cause, or for patients with subacute or chronic anemia, including patients with cancer, patients hospitalized in intensive care units or patients with terminal renal failure. For this systematic review, the population of interest will be patients (in-hospital or outpatient) with any medical condition requiring at least one RBC unit. There will be no age restriction for this systematic review.

#### Intervention (exposure)

RBC transfusion accounts for the major part of blood supply organizations blood product manufacture activities [16-18]. A RBC unit comes from a unique donor, whereas plasma and platelets may come from several different donors and be pooled into a single unit before transfusion. Since RBC products are the most commonly used blood product, have the most important societal and clinical outreach, and a single unit comes from a single donor, our review will focus on RBC transfusions only. We will include all studies evaluating donor characteristics, focusing on age, sex and blood group that assess at least one pre-defined donor characteristic and its effect on recipients.

#### Outcomes, setting and timeframe

The primary outcome for this review is mortality. However, to better appraise the impact of donor characteristics on outcome, we will not restrict outcomes in the search strategy. Rather, the search strategy will capture any clinical or surrogate outcomes related to donor characteristics. We will not use a limit for year of publication in this review.

#### Exclusion criteria

•Studies in which the study population includes non-transfused patients and from which it is impossible to obtain results for transfused patients.

•Studies with no measure of association between donor characteristics and transfusion outcome.

•Case reports (≤2 cases) and case series.

•Duplicates or ‘sub-cohorts’ of already published cohorts.

### Analysis plan

A descriptive analysis of all included studies will be first performed in tables and text form. Clinical, demographic, and methodological quality characteristics and results within study will be reported and discussed. An in-depth discussion of heterogeneity between studies will be provided where applicable. We will perform meta-analyses of suitable studies and provide a discussion of the results.

### Study characteristics

Each study will be categorized as observational or interventional. Interventional studies will be categorized as randomized or non-randomized, and observational studies will be further categorized as cohort (retrospective or prospective) and case-control studies. Further major characteristics of the study design will be reported. We will describe the population studied including the total number of patients, clinical characteristics, the number of transfused patients, the blood products transfused with the proportion for each blood product, the characteristics of included patients (age, gender and reasons for transfusion), and inclusion and exclusion criteria.

### Risk of bias

We expect that most of included studies will be observational. Meta-analyses of observational studies may be more prone to bias [16]. Particular care will be taken to adequately investigate the methodological and clinical risk of bias before proceeding with the meta-analysis.

### Methodological quality assessment

The methodological quality of the included studies in this systematic review will be evaluated by two independent reviewers using the Downs and Black tool for assessing risk of bias [15]. The Downs and Black tool is the updated evidence-based quality tool for use in assessing risk of bias when including non-randomized studies in systematic reviews of interventions recommended by the Cochrane Collaboration [16]. Specific coding instructions adapted for this review will be included for reviewers and will be piloted prior to implementation on all selected references. Overall and study-specific appraisal of methodological strengths and weaknesses will be reported.

### Clinical risk of bias and effect modification

There is a high risk of clinical heterogeneity introduced by the many potential indications of transfusions of RBCs. The studied populations will most likely be very different between studies. We will take appropriate care to adequately define the clinical characteristics of the transfused patients and the indication for transfusion and if possible contact authors for further clarification. Sensitivity analyses will be performed to evaluate impact of these differences.

Changes in manufacture and storage policies, as well as donor inclusion and exclusion criteria changed significantly over the years. For example, age limits for blood donation have increased in many jurisdictions [17,18], preservative solutions used for RBCs have changed and the manufacture process has been modified by performing universal leukoreduction on RBC products [1,4]. In cases where the exact donor inclusion criteria and manufacture characteristics are not reported, we will attempt to contact authors for further details.

### Primary analysis

For our primary outcome of mortality, we will provide for each of the donor characteristics the reported number of alive and dead patients for exposed and non-exposed patients. We will also report the number of patients lost to follow-up to assess the completeness of the reported events. We will collect adjusted mortality risk as odds ratios or relative risks if reported. When appropriate, we will provide pooled effect estimates as pooled log-odds ratios with 95% confidence intervals using a random-effects modeling approach. The results will be presented in tables and forest plots. Continuous endpoints will be described and pooled using weighted means ratios with random-effects modeling. Statistical heterogeneity will be reported using the I^2^ test with 95% confidence intervals. If the number of studies included is sufficient (≥10), we will investigate the presence of publication bias using funnel plot techniques.

### Planned subgroup analyses to explain clinical heterogeneity

Given that our study has the potential, by design, to include different populations that have received RBC transfusions, clinical heterogeneity is expected. To explore clinical and statistical heterogeneity, assuming that pooling of data is possible and appropriate, we will report pooled log-odds ratio for the following subgroups: 1) adults ≥18 years versus mixed children/adults, only children versus only adult, and we will consider subgroups among children (<1 month and neonates versus other children <18 years; 2) hospitalized patients versus outpatient transfused patients; 3) patients transfused in the intensive care units versus hospitalized but not in the intensive care units; 4) significant changes in donor inclusion criteria or manufacture strategy; 5) surgical versus medical population (≥75% of included patients); 6) patients with acute versus chronic anemia; and 7) continent where the studies were conducted (North America, South America, Europe, Asia, Australia and Oceania).

### Planned sensitivity analyses to investigate impact of study design

We will conduct subgroup analyses to explore and explain heterogeneity in effects sizes and investigate robustness of our results. It is impossible to identify all potential sensitivity analyses, but we can pre-specify important sensitivity analyses based on the Downs and Black tool for the quality assessment of randomized and non-randomized studies that will have to be performed: selection bias, comparability of groups, and appropriateness of outcome assessment (blinded, length of follow-up, complete follow-up) [15].

### Quality of evidence

The quality of the evidence obtained from this systematic review will be influenced by the different designs of included studies. Two reviewers will evaluate the quality of evidence for our primary outcome (mortality) according to five domains: study limitations, inconsistency of results, indirectness of evidence, imprecision and reporting bias, using the GRADE methodology [19]. We will report the quality as very low, low, moderate or high.

## Discussion

The rigorous systematic review methodology used for this project will ensure the best available knowledge synthesis on this topic. The internal peer-review and *a priori* submission of the protocol to PROSPERO will reduce the risk of bias of this review and ensure a transparent process throughout the project. The internal validation and piloting undertaken at every step of the review will decrease the risk of selection bias and systematic extraction errors. We will pilot and then use an evidence-based tool for the evaluation of methodological quality and will use the results for pre-specified subgroup analysis to explore potential methodological cause for heterogeneity.

The results from a systematic review are dependent on the quality of the underlying primary studies conducted. For the current review, we are aware that most, if not all studies will be cohort or case-control studies, and many will be retrospective. As such, we decided purposively to be very inclusive in our eligibility criteria so we can better appraise the extent of the available literature as we hypothesize that most of the evidence regarding the association between donor characteristics and transfusion outcomes may be of low methodological quality. Such a comprehensive approach will allow us to assess the potential impact of low-quality versus high-quality studies on the estimation of the observed associations.

The results of this systematic review will provide the best evidence regarding the potential clinical effects of donor characteristics on transfusion recipients to better guide policy and clinical practice. Our systematic review will synthesize and update current knowledge regarding evidence pertaining to blood donor selection. In addition, the results of our systematic review will identify clinical and methodological strengths and weaknesses of existing evidence regarding donor characteristics and recipient outcome. It will serve as a foundation for further research in the field and for the development of studies evaluating present or establishing novel policies regarding outcome improvement in transfusion medicine. The results of our work will allow blood collection and supply organizations to ensure that their current policies are evidence-based and could result in updating national policies regarding donor selection for blood transfusion. The potential optimal identification of donor characteristics that are associated with better RBC transfusion outcome may ultimately lead to an improvement in health outcomes in the transfused patient population.

## Competing interests

The authors declare that they have no competing interests.

## Authors’ contributions

MC and DF designed the review, conducted the scoping searches, drafted and revised the manuscript. SE, LM, GK, NS, AF, KW, CvW and AT provided content expertise, feedback on the design of the review, the protocol and on the manuscript. MC, DF and SE will be the reviewers for the systematic review. All authors read and approved the final manuscript.

## Supplementary Material

Additional file 1 Search strategy. Click here for file
