# Supplementary material for: Effect of blood donor characteristics on transfusion outcomes: a protocol for systematic review and meta-analysis
Source: Syst Rev. 2014 Mar 20;3:28. doi: 10.1186/2046-4053-3-28 (PMC3998188; doi:10.1186/2046-4053-3-28)
Supplement: Additional file 1 — Search strategy. [file 2046-4053-3-28-S1.doc]

# Appendix 1: Search strategy

Database: Embase Classic+Embase <1947 to 2013 December 11>, Ovid MEDLINE(R) In-Process & Other Non-Indexed Citations and Ovid MEDLINE(R) <1946 to Present>

Search Strategy:

--------------------------------------------------------------------------------

1 blood transfusion/ or erythrocyte transfusion/ (168687)

2 ((rbc or red blood$ cell$ or red cell$ or erythrocyte$) adj5 transfusion$).tw. (18530)

3 1 or 2 (175580)

4 Blood Donors/ (46008)

5 ((rbc or blood or red cell$ or erythrocyte$) adj1 (donor$ or donation$)).tw. (53965)

6 4 or 5 (72574)

7 3 and 6 (16580)

8 (risk or mortality).mp. or cohort.tw. (5021473)

9 prognosis/ or treatment outcome/ (2048249)

10 predictor$.tw. (498103)

11 diagnosed.tw. (854089)

12 exp models, statistical/ (397700)

13 death/ (185240)

14 or/8-13 (7507718)

15 7 and 14 (5724)

16 15 use prmz (1893)

17 *blood transfusion/ (70716)

18 *erythrocyte transfusion/ (6471)

19 ((rbc or red blood$ cell$ or red cell$ or erythrocyte$) adj5 transfusion$).tw. (18530)

20 17 or 18 or 19 (88179)

21 *Blood Donors/ (20782)

22 ((rbc or blood or red cell$ or erythrocyte$) adj1 (donor$ or donation$)).tw. (53965)

23 21 or 22 (59904)

24 20 and 23 (8332)

25 (risk or mortalit$ or cohort).tw. (3978080)

26 follow-up.mp. or prognos$.tw. or ep.fs. (4675345)

27 25 or 26 (7304408)

28 24 and 27 (3007)

29 28 use emczd (1814)

30 16 or 29 (3707)

31 remove duplicates from 30 (2993)

Cochrane Library

Description:

--------------------------------------------------------------------------------

ID Search

#1 MeSH descriptor: [Blood Transfusion] this term only

#2 MeSH descriptor: [Erythrocyte Transfusion] this term only

#3 rbc near/1 transfusion:ti,ab,kw or "red blood cell* NEAR1 transfusion":ti,ab,kw or red cell near/1 transfusion:ti,ab,kw or erythrocyte near/1 transfusion:ti,ab,kw (Word variations have been searched)

#4 platelet* near/1 transfusion:ti,ab,kw (Word variations have been searched)

#5 #1 or #3 or #4

#6 MeSH descriptor: [Blood Donors] explode all trees

#7 rbc near/1 (donor* or donation*):ti,ab,kw or "blood" near/1 (donor* or donation*):ti,ab,kw or "red cell" near/1 (donor* or donation*):ti,ab,kw or "erythrocyte" near/1 (donor* or donation*):ti,ab,kw (Word variations have been searched)

#8 #6 or #7

#9 #5 and #8

Cochrane Reviews (9)

Trials (130)

Technology Assessments (3)

Economic Evaluations (22)
